# Effect of Hydrologic Alteration on the Community Succession of Macrophytes at Xiangyang Site, Hanjiang River, China

**DOI:** 10.1155/2017/4083696

**Published:** 2017-01-03

**Authors:** Na Yang, Yehui Zhang, Kai Duan

**Affiliations:** ^1^College of Hydrometeorology, Nanjing University of Information Science & Technology, Nanjing 210004, China; ^2^State Key Laboratory of Water Resources and Hydropower Engineering Science, Wuhan City 430072, China; ^3^North Carolina State University, Raleigh, NC, USA

## Abstract

With the intensification of human activities over the past three decades in China, adverse effects on river ecosystem become more serious especially in the Hanjiang River. Xiangyang site is an important spawn ground for four domestic fishes in the downstream region of Hanjiang River. Based on the field survey results of macrophytes during 1997–2000 and 2013-2014, community succession of aquatic macrophytes at Xiangyang site was evaluated and discussed. Two-key ecologic-related hydrologic characteristics, flow regime and water level, were identified as the main influence factors. The EFC (environmental flow components) parameters were adopted to evaluate the alteration of flow regimes at Xiangyang site during 1941–2013. Evaluation results demonstrate a highly altered flow process after being regulated by reservoir. The flow patterns tend to be an attenuation process with no large floods occurring but a higher monthly low flow. Furthermore, the water level decreased and fluctuation reduced after the dam was built, which caused the decrease of biomass but favored the submerged macrophytes during 1995–2009. However, with the water level increasing after 2010 and gently fluctuating, due to uplift by the hydraulic projects downstream as well as the flow attenuation, the dominant position of submerged macrophytes will be weakened.

## 1. Introduction

The benign reproductive of aquatic macrophytes is increasingly recognized as a crucial factor to the aquatic ecosystem health and the food chain integrity [1]. Aquatic macrophytes influence many aspects of river structure and function, and their developmental changes strongly affect the whole river ecosystem process [2, 3]. Meanwhile, macrophytes abundance and community composition can be useful indicators of aquatic ecological health, as they reflect the quality of both physical and chemical habitat conditions [4, 5]. With the intense influences of human activities and climate change, rivers throughout world are at risk for loss of macrophytes communities and community succession [6]. Many surveys and experiments have been carried out to evaluate the status of aquatic macrophytes and try to find the main influence reasons, including illumination, nutrition, sediment, flow, water level, and temperature [7–10]. But few concerns focused on the change of natural characteristics of river based long-term historical data. Studying the evolution of natural hydrologic regime will probably reveal the external reason of degradation of macrophytes and communities succession [11, 12].

Hydrology is considered to be the most important determining factor in wetland functions [13]. Hydrological characteristics, such as water level and water flow, are significant factors influencing macrophytes [14]. Based on the studies on aquatic habitats, including macrophytes and fishes, Wantzen et al. [15] concluded that aquatic macrophytes growing in the littoral zone were sensitive to changes in the water level fluctuation regime. Ogdahl and Steinman [5] surveyed macrophytes beds in Muskegon Lake over a 4-year period to assess the ecological benefits of the restoration project and found that macrophyte biomass was affected strongly by the physical features of the individual sites (i.e., hydrologic exposure) and a possible environmental cause (i.e., water level and air temperature). Mjelde et al. [14] developed a water level drawdown index for Nordic lakes to identify the suitable fluctuation amplitude range for the sensitive and tolerant aquatic plants. Zhu et al. [16] conducted an experiment to investigate the plant growth, root anchorage strength, and stem tensile properties of five submerged macrophytes under three initial water levels (1.0, 2.5, and 4.0 m) with four water level fluctuation speeds (0, 5, 15, and 25 cm d^−1^). The results showed that deep water can inhibit plant growth and decrease their mechanical resistance. All of those studies demonstrated an important role of hydrologic characteristics in the growth of aquatic macrophytes. However, it is worth noting that those studies were based on a short-term series, normally a few months or 4-5 years, which was too short to evaluate the change of hydrologic characteristics. The hydrological characteristics around the world present complex changes due to human disturbance, such as reservoir operation, water diversion, and land appropriation [17–19] that require long history data to evaluate. In this paper, long-term hydrologic data between 1943 and 2014 were used to evaluate the river ecosystem-related hydrologic alteration and reveal the reasons of community succession of Xiangyang site, which is the important spawn ground of the four domestic fishes in Hanjiang River. The paper is organized as follows: Section 2 introduces the data and methodology used for evaluation; Section 3 presents the evaluation results of community succession and hydrological alteration; Section 4 conducts the discussion of the influences of hydrological alteration; the conclusions are summarized in Section 5.

## 2. Study Area, Data, and Methods

### 2.1. Study Area

Xiangyang site is located at the mid reach of Hanjiang River with a humid, monsoon climate. The average annual precipitation, temperature, and runoff during the period of 1960–2013 were 865 mm, 16°C, and 1197 m^3^/s, respectively. About 85% of the annual runoff comes from the Han River, with the rest from the South River, North River, Tanghe River, and Xiaoqing River. The current aquatic macrophytes communities at Xiangyang site include* P. perfoliatus, P. malaianus, Vallisneria natans, Salvinia natans, A. philoxeroides, *and* Artemisia selengensis* [20]. Since 1960s, the river flow of Xiangyang site is greatly regulated by the Danjiangkou reservoir in the upstream region, which is a large-comprehensive reservoir and required to supply water for the mid-route of South-to-North water diversion project in 2014. With more water stored and transferred from the upstream Hanjiang River, the hydrological characteristics of Xiangyang site have been greatly changed, which boost the pressure of local water management and ecological restoration. It is necessary to study the hydrological alteration and evaluate the aquatic ecosystem health to provide scientific basis for the protection of river ecosystem and sustain the function of spawning ground of four domestic fishes.

### 2.2. Data

In this study, the daily flow data of Xiangyang hydrological station were obtained from 1941 to 1960 and 1973 to 2013. The observed data during 1961–1972 was not available because Danjiangkou Dam was built and blocked the river. We also collected the 6-hour water level measurement data during 1956–1960 and 1973–2014; the missing data during a day are filled by using linear interpolation. Besides, the cross-section measurement data of Xiangyang site were obtained as well. All of those hydrologic data were excerpted from the “Hydrological Year Book” published by the Hydrological Bureau of the Ministry of Water Resources.

We also collected some measurement data about the aquatic macrophytes of Mid-Lower Hanjiang River from two field surveys conducted during 1997–2000 [20] and 2013-2014 (unpublished). During 1997–2000, three parallel areas were selected as sample site at the Xiangyang site with the sampling interval of 5 km. The covered distance from the center of each sample site was 1 km, during which the coastal area, subcoastal zone, central deep water area, and shoal were selected as the sampling sites. GPS was used for positioning. The aquatic plant communities in the shallow water area and the wetlands were collected by harvesting, and 2 m × 2 m grass samplers were chosen for recording. During 2013-2014, the plant communities were sampled with the methods of sampling in parallel and repeated small random sampling plots. The heights of the plant community above 2 m, 2 m-1 m, and 1 m were sampled at 2 m × 2 m, 1 m × 1 m, and 0.5 m × 0.5 m, respectively, and the minimum sampling area of each community was not less than 3 samples. The aquatic plant communities in the shallow water area and the river surface were collected by harvesting, and 0.5 m × 0.5 m grass samplers were used for the river surface sampling. All the plants in the quadrats were uprooted, washed, and weighed (wet weight). The two-field surveys revealed the changes of macrophytes types and distribution at Xiangyang site.

### 2.3. Environmental Flow Component

Not only is it essential to maintain adequate flows during low flow periods, but higher flows and floods and extreme low flow conditions also perform important ecological functions [21]. Ecological researchers have demonstrated that river hydrographs can be divided into a repeating set of hydrographic patterns that are ecologically relevant. Based on the widely used evaluation indexes of flow regime [22], five types of flow events were proposed to represent the full spectrum of flow conditions that are crucial to sustain riverine ecological integrity; they are low flows, extreme low flows, high flow pulses, small floods, and large floods [23]. The five EFC types are described in more detail in Table 1.

The five EFC types were classified based on the predam daily flow data series, which were regarded as the natural flow without human disturbance. We firstly defined the high flows and low flows, which were simply separated by using a sing fixed threshold. The flows that exceeded 75% of daily flow for the period will be classified as high flows, while those below this level will be classified as low flows. A small flood event was defined as an initial high flow with a peak flow greater than 2-year return interval event. A large flood event was defined as an initial high flow with a peak flow greater than 10-year return interval event. An extreme low flow was defined as an initial low flow below 10% of daily flows for the period.

## 3. Results

### 3.1. Community Succession of Macrophytes at Xiangyang Site

Based on the survey results, the aquatic macrophytes communities during 1997–2000 and 2013-2014 are evaluated in this section. It is clear that the major community types in the two periods are totally different (Table 2). During 1997–2000, there were two dominant plant communities, Ass.* P. pectinatus* +* P. perfoliatus* and Ass.* P. malaianus* +* Hydrocharis dubia*; both belonged to the submerged aquatic macrophytes. When coming to 2013-2014, the macrophyte communities of Xiangyang site changed to submerged plants, floating plants, emergent plants, and hygrophytes, respectively, represented by* P. malaianus *and* P. perfoliatus*,* Salvinia natans *and* V. natans*,* A. philoxeroides,* and* Artemisia selengensis*. It demonstrates the succession of aquatic macrophytes community occurring during 2013-2014 at Xiangyang site. According to their surveys in the whole Mid-Lower Hanjiang River, the ratio of submerged macrophytes association with the total population decreased from 70% in 1997–2000 to 31.58% in 2013-2014, and the ratio of hygrophytes association increased from zero to 31.58%. It seems that the community succession of macrophytes occurred in the whole Mid-Lower Hanjiang River basin, which also influences the macrophytes types of Xiangyang site. Moreover, the aquatic macrophytes of Xiangyang site were greatly degraded with the biomass declining from 12025 g/m^2^ to 7789 g/m^2^. The community succession and degradation of macrophytes strongly indicate the evolution of the river ecosystem, which is probably caused by human disturbance and the change of natural environment.

**Figure 1:**
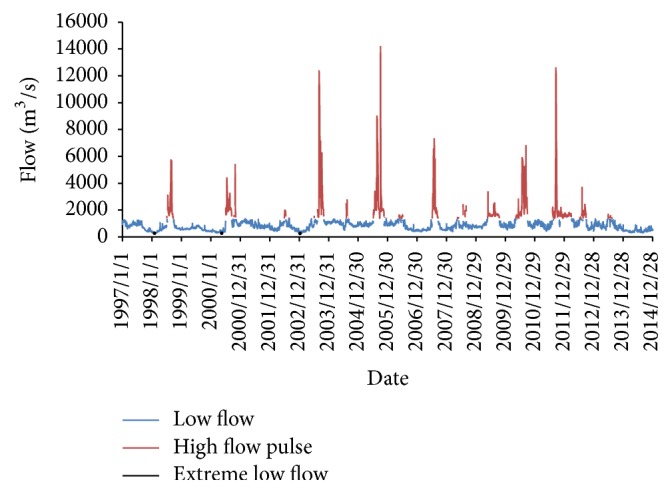
The environmental flow component of Xiangyang site during 1997–2014.

To assess the influence of hydrologic alteration, we calculate the EFC of Xiangyang site during 1997–2014 based on the daily flow data. The thresholds of low flow, high pulse flow, extreme low flow, small foods, and large floods were all determined by the predam observed daily data, which was regarded as the natural regime. Form Figure 1, we clearly see that no flood events were observed during the whole period of 1997–2013, which indicates a smaller and smoother flow regime compared with the natural flow. Figure 1 also demonstrates a smaller low flow and a less occurrence of high flow pulse during 2013-2014 compared with 1997–2000. For the period 2013-2014 is shorter than 1997–2000, we calculated the occurrence proportions of the three flow events during each period. The results show that between 1997 and 2000, the high flow pulse accounted for 11% of the whole flow events, while in 2013-2014 the occurrence proportion of high flow pulse was just 5%. However, the occurrence proportion of low flows was increased from 88% during 1997–2000 to 95% in 2013-2014, and no extreme low flows were observed in 2013-2014. It seems that the river flow during 2013-2014 was more flat with a relative smaller volume and fluctuations, which is more favorable for those low flow-enduring aquatic plants and small fluctuation-appetite plants.

**Figure 2:**
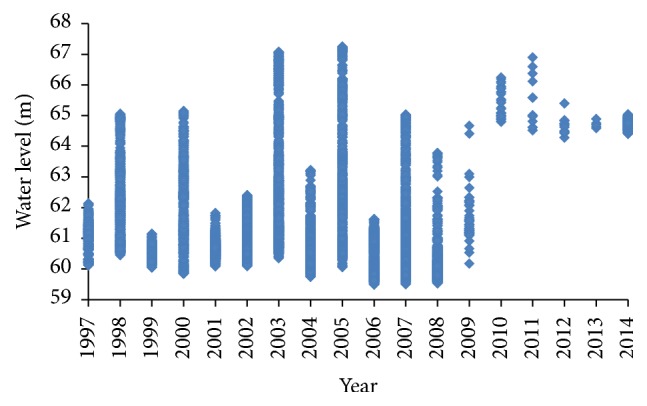
The distribution of water level within a year during 1997–2014.

The water level fluctuation is also very important with regard to affecting the function of river ecosystem, especially in the growth and distribution of aquatic plants [24, 25]. Based on the water level measurement data between June and October during 1997–2014, we surprisingly find that the water level during 2013-2014 presented a quite flat and high level (Figure 2). The water level of 2013-2014 maintained a high level of above 64.5 m, much higher than the average water level during 1997–2000. Zhu et al. [16] found that the submerged macrophytes are inadaptable to deep water level that their biomass, relative growth rate, and roots anchorage strength decreased with increasing initial water level. Moreover, with the water level increasing, the illumination decreases. Changes in light intensity and nutrient concentrations were considered to be key determinants of growth of submerged macrophytes [26, 27]. This partly explains the decrease of submerged macrophytes during the high water level period of 2013-2014. Besides, the fluctuation range of water level was significantly reducing. Before 2010, the fluctuation range of water level was large with the maximum fluctuation amplitude of 6.74 m occurring in 2005 and average water level fluctuation amplitude of 2.98 m. However, the fluctuation amplitude reduced suddenly after 2009 with a value of 0.3 m in 2013 and 0.62 m in 2014. The reduced fluctuation of water level probably promotes the growth of some aquatic macrophytes.

As an important factor of human disturbance, water quality determines the sound growth of aquatic plants in many aspects. According to the agriculture information data from the local government in 2000 and 2014, the NH_4_-N of Xiangyang site decreased from 0.256 mg/L to 0.248 mg/L, the TN increased from 1.39 mg/L to 1.74 mg/L, and the TP increased from 0.034 mg/L to 0.059 mg/L. The change of chemical concentrate was not significant in the 14 years compared with the change of hydrologic characteristics. Consequently, a conclusion can be preliminarily derived where the macrophytes degradation and community succession at Xiangyang site occurred. It is strongly related to hydrologic alteration, such as a smaller flow and a less fluctuation of water level, which may change the types and distribution of aquatic macrophytes and influence the effectiveness of river ecological restoration. Accordingly, it is necessary to deeply evaluate the hydrologic alteration at Xiangyang site so as to provide more scientific suggestions for river ecosystem restoration.

### 3.2. The Hydrologic Alterations

To evaluate the change of hydrologic characteristics, we firstly divide the study period into predam periods (1941–1960) and postdam periods (1974–2013) based on the daily flow data of Xiangyang hydrological station. And then we separate the postdam period into two stages: the less human disturbance stage (1973–1990) and the intense human disturbance stage (1991–2013).

According to the EFC assessment frame, we calculated the 32 EFC parameters in the predam periods and postdam periods, respectively, listed in Table 3. It is notable that the length of observed flow data in the predam period is less than 20 years that some information will be lost when calculating the frequency of flood events. For example, the frequency of large food in predam period is nearly 0 but actually happened 4 times. Therefore, we substitute counts for the frequency to reveal the happening of the flood events. From Table 3, we clearly find that, compared with the predam period, all of the 32 parameters changed in different degrees with the large flood events changing mostly. Since the Danjiangkou Dam was built, no large flood events happened in terms of the zero values of all the six parameters in the large floods group, although the length of postdam period is longer than predam period. On the other side, the low flow of the 12 months all became bigger than in predam period in terms of the positive relative alteration rate. It is worth noting that the largest relative alteration rate all happened in low flow seasons, such as January, February, and December. It indicated a bigger flow process in dry seasons than predam period. In addition, significant decrease is seen clearly in the frequency and duration of extreme low flows (RB = −0.77 and −1, resp.). The flow peak, duration, frequency, rising rate, and falling rate of high flow pulse all presented decreasing trend with the rising rate and falling rate decreasing most drastically (RA = −0.83 and −0.73, resp.). The small flood counts greatly decreased compared with predam period (9 versus 42) even though the postdam period was much longer than the predam period. The flow regime alteration after dam was built demonstrates that the river flow of Xiangyang hydrological station behaved in a homogenization and attenuation tendency.

It is notable that most of the parameters with significant change primarily happened during 1991–2013, the intense human disturbance period, such as the January low flow, February low flow, December low flow, the extreme low flow counts, the rise and fall rate of high flow pulse, and the decrease of small flood events. The relative alterations of low flow in January, February, and December were much higher during 1991–2013 with the values of 1.13, 1.22, and 0.66, compared to the relative alteration of 0.13, 0.09, and 0.09 during 1974–1990. And the relative alteration rate of rise rate of high flow pulse during 1991–2013 was high up to −0.81 compared with −0.12 during 1973–1990, similar to the fall rate of high flow pulse. What is more, the small floods in the postdam period all happened before 1990. No floods were observed during 1991–2013. It indicates a more significant change of flow regime after 1990 under the impact of intense human activities.

**Figure 3:**
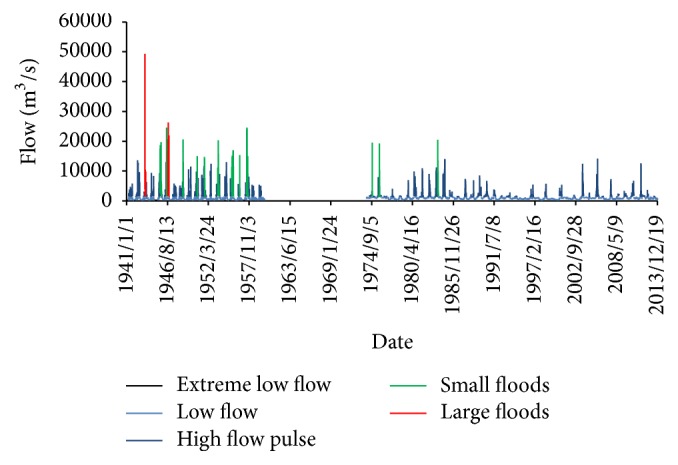
The results of environmental flow component (EFC) from 1941 to 2013.

Figure 3 shows the spectrum of environmental flow component from 1941 to 2013 (except the missing data), which clearly demonstrates a smaller flow process and the attenuating fluctuation after dam was built. Compared with the predam period, the large floods totally disappeared and the small floods were decreased obviously in magnitude and occurrence. The peak of high flow pulses became smaller and low flows became larger. Besides, Figure 3 also shows a substantial change of flow in the intense human disturbance period, such as the disappearing of small foods and low occurrence of high flow pulse.

Accordingly, it is reasonable to believe that the flow regime of Xiangyang hydrological station has been changed to a great degree since dam was built, especially after 1990. The disappearing of flood events was largely caused by the flood control in flood seasons, and the higher monthly low flows were mainly due to the water supply from reservoir in nonflood seasons. The reduced fluctuation of high flow pulse was because of setting reservoir regulation rules formulated by the government. Those great changes in flow regime could be a trigger of changing in the structure of aquatic and riparian macrophytes.

### 3.3. The Water Level Variation

**Figure 4:**
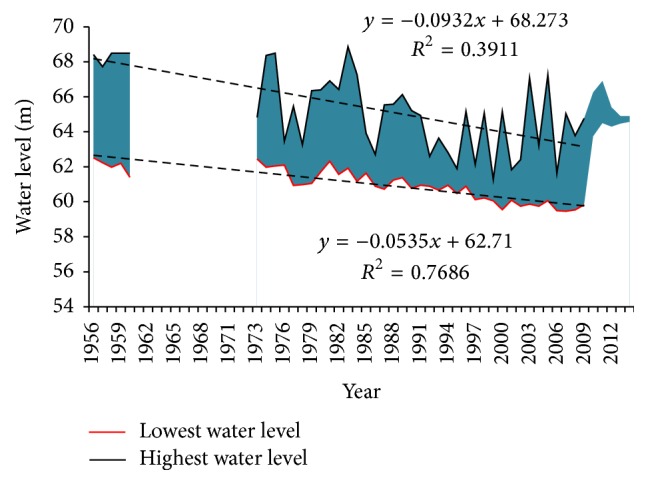
The fluctuation range of water level at Xiangyang site between 1973 and 2014.

Water level is changing with time but has a stable statistical fluctuation path when seeing a long-term measurement data, which is favorable for maintaining the stability of the aquatic plants structure. However, with the change of flow regime and human disturbance, the law of water level fluctuation changed as well.  Figure 4 shows the time series of water level fluctuation range of Xiangyang hydrological station from May to October during 1956–2014, the aquatic plant growing period. Due to the same reason, the observed data of water level was missing during 1961–1973. It is clearly seen that both of the two time series present significant decrease during 1973–2009 with the lowest water level decreasing more significantly. Meanwhile, the fluctuation range presented reducing trend compared with the predam period. The decrease of water level and fluctuation range was related to the flood attenuation caused by flood control. What is more, the changing in physical features of the river channel also led to water level decreasing.

Xiangyang is located 109 km away from the Danjiangkou Dam. Affected by the damming upstream, the sediment source running into the downstream channel was changed radically. In the predam period, the sediment in Xiangyang section was basically brought by upstream runoff. But now, due to reservoir, the sediment from upstream regions is zero if without reservoir flood discharge in nonflood seasons, and only 1.98% of reservoir sediment will be deposited into the downstream channel with the flood discharge. Instead, the sediment in the downstream river channel mainly comes from the limited silt scored from the river bed or brought by the tributary. It means that the sediment of Xiangyang site is decreasing year by year and the inflow become much cleaner than before. With the scouring of clean water and less sedimentation, the channel physical characteristics were changed to some degree, and the relationship between water level and river flow was changed correspondingly.

**Figure 5:**
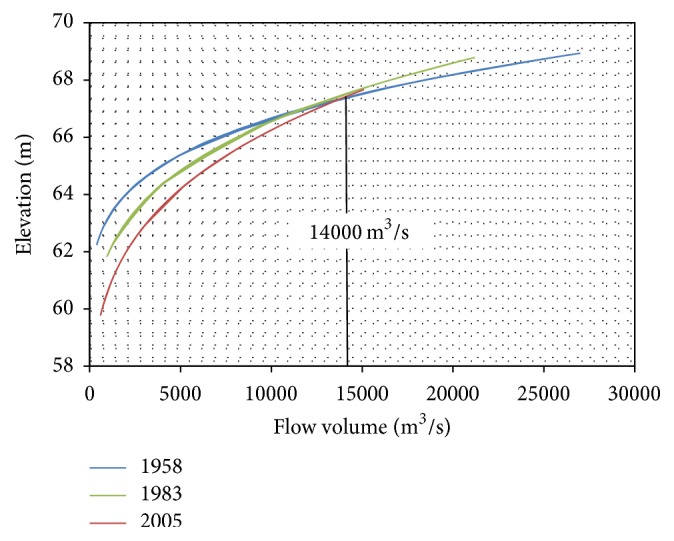
The relation curves of water level and flow (*Z*~*Q*) of the three periods.

Figure 5 shows the *Z*~*Q* relation curves of the three years, 1958, 1983, and 2005. It is clear that the three relation curves, respectively, reflect the changes in the shape of river channel in different periods. Hereinto, the relation curve of 1958 represents a natural state of river channel without human disturbance. Under the impact of reservoir operation, the *Z*~*Q* relation behaved differently with the change of sediment component-amount and developed to a new stability after 1983, when large flood happened and created a strong scour. After 1983, the downstream channel was experiencing the clear water scour. The different behaviors of the three relation curves lead to the same flow volume producing different water levels in different periods. When the river flow was smaller than 14,000 m^3^/s, the water level in 1958 behaved highest with the same amount of inflow, while the water level of 2005 behaved lowest. The results were totally inverted when the river flow was greater than 14,000 m^3^/s. Based on the flood data, the observed value of flood peak dated from 1984 was rarely greater than 14,000 m^3^/s. It seems that the river channel was less and less submerged after dam was built even with the same amount of released water. The truth is that released water was greatly reduced that the study river was experiencing a lower water level period in the postdam period. With the decreasing of water level, some low water level-adaptable macrophytes probably started to grow and develop, such as submerged macrophytes. However, the water level rose suddenly after 2009, which was caused by the flow uplift of downstream hydraulic project. The water level rising further changed the relation curve of *Z*~*Q* and the macrophytes structure of Xiangyang site.

Based on the evaluation of hydrologic alteration on the flow regime and water level, it is reasonable to believe that the hydrologic features of Xiangyang site have been greatly changed due to reservoir operation and human disturbance. The river flow tended to be smaller and the water level became lower compared with the predam period. The sudden rise of water level from 2010 was caused by the uplift of downstream hydraulic project, which will continue to raise the water level of Xiangyang site in the future. Those changes imposed great influences on the adjustment of aquatic macrophytes structure.

## 4. Discussions

The study of fresh wetland indicated that productivity and decomposition of aquatic species are positively related to the magnitude of hydrologic inputs [28, 29]. The average gross primary productivity of water column producers in the high flow wetland was generally higher than in low flow wetlands [30]. Compared with predam period, a relative smaller flow process was observed in the aquatic plant growth period (generally between April and October), in terms of the disappearing of large floods and significant decrease of small floods, which partly hindered the growth of aquatic plants and led to the decreasing of biomass at Xiangyang site. Meanwhile, the changing in relation curve of *Z*~*Q* leads to an even lower water level with the regulated smaller flow input and aggravated the decrease of biomass. According to the water level measurement data from 1956 to 2014, the minimum water level decreased from 62.51 m in 1956 to 59.83 m in 2009, and the maximum water level decreased from 68.41 m in 1956 to 64.76 m in 2009 (see Figure 4). The average decrease rate was about 0.53 m/year and 0.73 m/year, respectively. The shallow zone will be totally exposed when the water level is lower than 59.49 m based on the cross-section of Xiangyang site in 2006. The decrease of water level and smaller inflow resulted in the river channel being less submerged that some part of the river channel was not suitable for the growth of aquatic plants. Accordingly, we speculate that the macrophytes biomass of Xiangyang site presented decreasing trend over a long-term period after dam was built.

**Figure 6:**
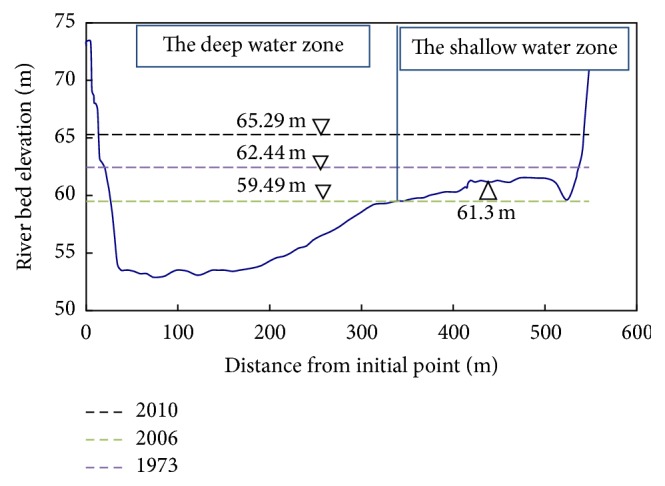
The cross-section of Xiangyang site and the minimum water level in 2010, 2006, and 1973.

However, the lower water level could provide the ideal growth condition for the submerged aquatic plants. Zhu et al. [16] investigated the lake in Yunnan province, located in the southwest of China, and found that the uppermost depth for most submerged plants was approximately 6 m and the highest biomass of an individual plant was within 3 m. From 1973 to 2006, the minimum water level decreased from 62.44 m to 59.49 m, with the corresponding water depth decreasing from 0.08–9 m to 0–6 m based on the cross-section of Xiangyang site (Figure 6). It seems that the water level of the whole section was more suitable for submerged macrophytes during 1995–2006 when there was a lower water level, while only some shallow zones were suitable for the submerged plants in the early period. The phenomenon of submerged macrophytes being the dominant species during 1997–2000 was largely related to the suitable water level.

But the sudden rise of water level after 2009 imposes an unfavorable influence on the growth of aquatic plants. This is because deep water decreases light availability that hinders the growth of aquatic plants [31]. After 2010, the minimum water level rose to 65.29 m sharply in 2010 with a water depth of 11.29 m, which exceeded the uppermost depth of the submerged plants and reduced the growth rate of emergent plants. But the shallow zone was totally submerged again with the water level rising, with a depth of approximately 3 m according to the cross-section. It was the most appropriate living environment for submerged macrophytes. It explains why the submerged plants were just degraded but not disappearing from the macrophyte communities of Xiangyang site after the water level rose to a pretty high level. Considering the geometry of the cross-section of Xiangyang site and irrevocable influences from hydraulic project, the water level within 62.3–65.3 m is appropriate to keep the shallow water zone submerged and make it suitable for the growth of submerged macrophytes. On the other side, after the water level rising, the stable water level in the shallow water zone also provided more chances for the leaves surfacing, which favored the growth of emergent macrophytes and floating plants [32]. It seems that the dominant position of submerged plants will be weakened to a certain degree in the future. It is noteworthy that, influenced by the continuous uplift of downstream hydraulic project, the flow velocity is decreased and the water level fluctuation is reduced, against the water self-purification and pollutant output. The water quality should be highly valued to prevent the deterioration of the living circumstances of submerged plants.

## 5. Conclusions

In this study, the succession of macrophytes communities at Xiangyang site is discussed. Two ecology-related hydrological features at Xiangyang site, flow regime and water level, are evaluated into three periods based on their time sequence characteristics and the Hanjiang River development. The main reasons of changing in those parameters are discussed and explained. Some main findings can be summarized as follows:

(1) The status of macrophyte communities at Xiangyang site is evaluated by comparing the survey results during 1997–2000 and 2013-2014. The results demonstrate an obvious macrophytes succession in terms of the submerged plants disappearing and new species appearing. The macrophytes types changed from submerged macrophytes to a diversity pattern including submerged plants, emergent plants, hygrophyte, and floating plants. And the biomass declined from 12025 g/m^2^ to 7789 g/m^2^, which indicates the community degradation at Xiangyang site. The main reasons were identified as hydrologic alteration.
(2) The flow regime of Xiangyang site has been greatly changed due to dam being built with the flood events decreasing most significantly. Moreover, the monthly low flow presented increase in dry seasons and the fluctuation of high flow pulse became smaller. The variability of flow regime indicates a homogenization and attenuation tendency. The alteration of flow regime behaved most drastically during 1991–2013, which was influenced by the intense human activities in the end of last century. The smaller flow input partly resulted in the decrease of biomass at Xiangyang site.
(3) The variability of water level during 1973–2013 is evaluated and the results demonstrate a downward trend and a decreased fluctuation range. Beside impact of the smaller flow input, the changing in water level variation was also related to the long-term clear water scour and less sedimentation that changed the physical characteristics of the river channel. The relation curve between water level and river flow (*Z*~*Q*) after dam was built was changed to a low efficient pattern. The same flow volume corresponded to a lower water level in recent period compared with the predam period. It indicates that in most time of postdam period the channel was less submerged which makes it possible for the growth of some shallow water adaptable macrophytes.
(4) The influences of long-term hydrologic alteration on the community succession and macrophytes degradation of Xiangyang site are discussed. The deceasing water level promoted submerged plants being the dominant plants during the field survey period of 1997–2000, while the water level sudden rise after 2009 caused the disappearing of submerged plants in the deep water zone. However, the shallow water zone was totally submerged which reprovided favorable environment for the growth of submerged plants. The appropriate water level of Xiangyang site should be 62.3–63.5 m for the growth of submerged plants and maintaining the shallow water zone submerged.

Based on the evaluation of the influence of hydrologic alteration, we can conclude that hydrologic characteristics, especially water levels, play an important role in the growth of aquatic macrophytes. The restoration of river ecosystem should fully consider the change trend of hydrologic features and formulate more effective and feasible plans.

## Figures and Tables

**Table 1:** Summary of environmental flow component (EFC) parameters and their ecosystem influences.

EFC type	Hydrologic parameters	Ecosystem influences
(1) Monthly low flows	Mean or median values of low flows during each calendar month *(Subtotal 12 parameters)*	(1) Provide adequate habitat for aquatic organisms; (2) maintain suitable water temperatures, dissolved oxygen, and water chemistry; (3) maintain water table levels in floodplain, soil moisture for plants; (4) provide drinking water for terrestrial animals; (5) keep fish and amphibian eggs suspended; (6) enable fish to move to feeding and spawning areas; (7) support hyporheic organisms (living in saturated sediments)

(2) Extreme low flows	Frequency of extreme low flows during each water year or season Mean or median values of extreme low flow event Duration (days) Peak flow (minimum flow during event) Timing (Julian date of peak flow) *(Subtotal 4 parameters)*	(1) Enable recruitment of certain floodplain plant species; (2) purge invasive and introduced species from aquatic and riparian communities; (3) concentrate prey into limited areas to benefit predators

(3) High flow pulses	Frequency of high flow pulses during each water year or season Mean or median values of high flow pulse event Duration (days) Peak flow (maximum flow during event) Timing (Julian date of peak flow) Rise and fall rates *(Subtotal 6 parameters)*	(1) shape physical character of river channel, including pools, riffles; (2) determine size of streambed substrates (sand, gravel, cobble); (3) prevent riparian vegetation from encroaching into channel; (4) restore normal water quality conditions after prolonged low flows, flushing away waste products and pollutants; (5) aerated eggs in spawning gravels and prevent siltation; (6) maintain suitable salinity conditions in estuaries

(4) Small floods	Frequency of small flood during each water year or season Mean or median values of high flow pulse event Duration (days) Peak flow (maximum flow during event) Timing (Julian date of peak flow) Rise and fall rates *(Subtotal 6 parameters)*	Applies to small and large floods: (1) provide migration and spawning cues for fish; (2) trigger new phase in life cycle (i.e., insects); (3) enable fish to spawn in floodplain and provide nursery area for juvenile fish; (4) provide new feeding opportunities for fish, water flow; (5) recharge floodplain water table; (6) maintain diversity in floodplain forest types through prolonged inundation (i.e., different plant species have different tolerances); (7) control distribution and abundance of plants on floodplain; (8) deposit nutrients on floodplain

(5) Large flood	Frequency of large flood during each water year or season Mean or median values of high flow pulse event Duration (days) Peak flow (maximum flow during event) Timing (Julian date of peak flow) Rise and fall rates *(Subtotal 6 parameters)*	Applies to small and large floods: (1) maintain balance of species in aquatic and riparian communities; (2) create sites for recruitment of colonizing plants; (3) shape physical habitats of floodplain; (4) deposit gravel and cobbles in spawning areas; (5) flush organic materials (food) and woody debris (habitat structures) into channel; (6) purge invasive, introduced species from aquatic and riparian communities; (7) disburse seeds and fruits of riparian plants; (8) drive lateral movement of river channel, forming new habitats (secondary channels, oxbow lakes); (9) provide plant seedlings with prolonged access to soil moisture

**Table 2:** The Biomass and distribution of aquatic plant communities at Xiangyang site in different periods.

1997–2000			2013-2014		
Major community type	Biomass (g/m^2^)	Coverage (%)	Major community type	Biomass (g/m^2^)	Coverage (%)
(1) Ass. *P. pectinatus *+* P. perfoliatus* (2) Ass. *P*.* malaianus *+* Hydrocharis dubia*	12025	70	(1) Ass. *P. malaianus* + *P. perfoliatus* (2) Ass. *Salvinia natans* and *V. natans* (3) Ass. *A. philoxeroides* (4) Ass. *Artemisia selengensis*	7789	75

**Table 3:** Comparison results of the parameters of EFC in the three periods at Xiangyang site.

Parameters	Pre	Post			Relative alteration		
		Post (all)	Post (1974–1990)	Post (1991–2013)	Post (all)	Post (1974–1990)	Post (1991–2013)
January low flow	354	857	971	754	**1.42**	0.13	**1.13 **
February low flow	354	852	931	786.3	**1.4**	0.09	**1.22 **
March low flow	551	830	883	770	0.51	0.06	0.40
April low flow	724	853	936.8	853	0.18	0.10	0.18
May low flow	826	988	1040	893	0.2	0.05	0.08
June low flow	582	949	1160	889.3	0.63	0.22	0.53
July low flow	761	1130	1245	1125	0.48	0.10	0.48
August low flow	767	1175	1270	1090	0.53	0.08	0.42
September low flow	887	1050	1220	947.5	0.18	0.16	0.07
October low flow	785	790	1050	719	0.01	0.33	−0.08
November low flow	624	764	941.5	749.5	0.23	0.23	0.20
December low flow	422	746	810.5	700	**0.77**	0.09	**0.66 **

Extreme low peak	262	264	264	252	0.01	0	−0.04
Extreme low duration	11	2.5	4.5	1.75	**−0.77**	**0.80 **	**−0.84 **
Extreme low timing	45.5	48	60	22	0.05	0.25	−0.52
Extreme low counts	694	146	136	10	**−1**	−0.07	**−0.99 **

High flow peak	2430	1530	1530	1520	−0.37	0	−0.37
High flow duration	5	3	3.5	3	−0.4	0.17	−0.40
High flow timing	182	201	200	205	0.1	0.00	0.13
High flow frequency	9	6	8	4	−0.33	0.33	−0.56
High flow rise rate	758	125	110	145.5	**−0.83**	−0.12	**−0.81 **
High flow fall rate	−408	−110	−86.14	−126.2	**−0.73**	−0.22	**−0.69 **

Small flood peak	19700	19500	19400		−0.01	−0.01	/
Small flood duration	28	39	46		0.39	0.18	/
Small flood timing	218	278	278		0.28	0.28	/
Small flood counts	42	9	9	0	**−0.78**	**−0.78**	/
Small flood rise rate	1408	4830	3250		**2.43**	−0.33	/
Small flood fall rate	−800	−532	−1165		−0.33	**1.19 **	/

Large flood peak	37800				/	/	/
Large flood duration	49				/	/	/
Large flood timing	225.5		281		/	/	/
Large flood counts	4	0	0	0	/	/	/
Large flood rise rate	8814		4830		/	/	/
Large flood fall rate	−916		−532.2		/	/	/
